# Comparison of one-hole split endoscopic discectomy and microendoscopic discectomy in the treatment of lumbar disk herniation: a one-year retrospective cohort study

**DOI:** 10.1186/s13018-024-04574-6

**Published:** 2024-02-06

**Authors:** Chen Liu, Wencan Zhang, Chongyi Wang, Bingtao Hu, Kaibin Wang, Yunze Feng, Le Li, Wanlong Xu, Haipeng Si

**Affiliations:** 1 Department of Orthopedics, Qilu Hospital, Cheeloo College of Medicine, Shandong University, Jinan, 250012 Shandong People’s Republic of China; 2 Department of Orthopedics, Qilu Hospital (Qingdao), Shandong University, Qingdao, 266035 Shandong People’s Republic of China

**Keywords:** One-hole split endoscopic discectomy (OSE), Microendoscopic discectomy (MED), Lumbar disk herniation (LDH), Non-randomized clinical trial, Cohort study

## Abstract

**Background:**

We aim to compare and assess the surgical parameters and follow-up information of one-hole split endoscopic discectomy (OSE) and microendoscopic discectomy (MED) in the treatment of LDH.

**Methods:**

This study included 154 patients with degenerative lumbar disk disease. Sixty-eight patients underwent OSE and 86 patients MED. The VAS score for lower back and lower limb radiation pain, ODI score, modified MacNab score, estimated blood loss (EBL), length of the incision, amount of C-reactive protein, and recurrence and complication rates were examined as indicators for clinical outcomes and adverse events.

**Results:**

After surgery, the VAS and ODI scores in the two groups significantly decreased. On the third day after surgery, the VAS and ODI scores of the OSE group were significantly better than those of the MED group. The VAS and ODI scores preoperatively and at 1 month, 3 months, 6 months, and 12 months following the procedure did not substantially vary between the two groups. There was less EBL and a shorter incision with OSE than with MED. There was no significant difference in the rate of complications between the two groups.

**Conclusion:**

Compared with MED, OSE is a new alternative option for LDH that can achieve similar and satisfactory clinical outcomes. Furthermore, OSE has many advantages, including less EBL and a smaller incision. Further clinical studies are needed to confirm the effectiveness of OSE.

## Introduction

Lumbar disk herniation (LDH) is a common degenerative disease [1]. Since it was first introduced in the 1930s, open discectomy has continued to be the accepted method for treating LDH despite its disadvantages [2]. Currently, traditional open discectomy is being replaced with minimally invasive spine surgeries [3]. To enhance the clinical results and reduce the incidences of sequela and operation-induced trauma, minimally invasive surgery is used [4, 5]. Microendoscopic discectomy (MED) is one of the most popular minimally invasive spinal surgeries that has been used for the treatment of LDH [6, 7]. The advantages of this endoscopic surgery, such as its minimal trauma, rapid recovery, and straightforward procedure, have led to its widespread use in recent years [2, 8].

**Fig. 1:**
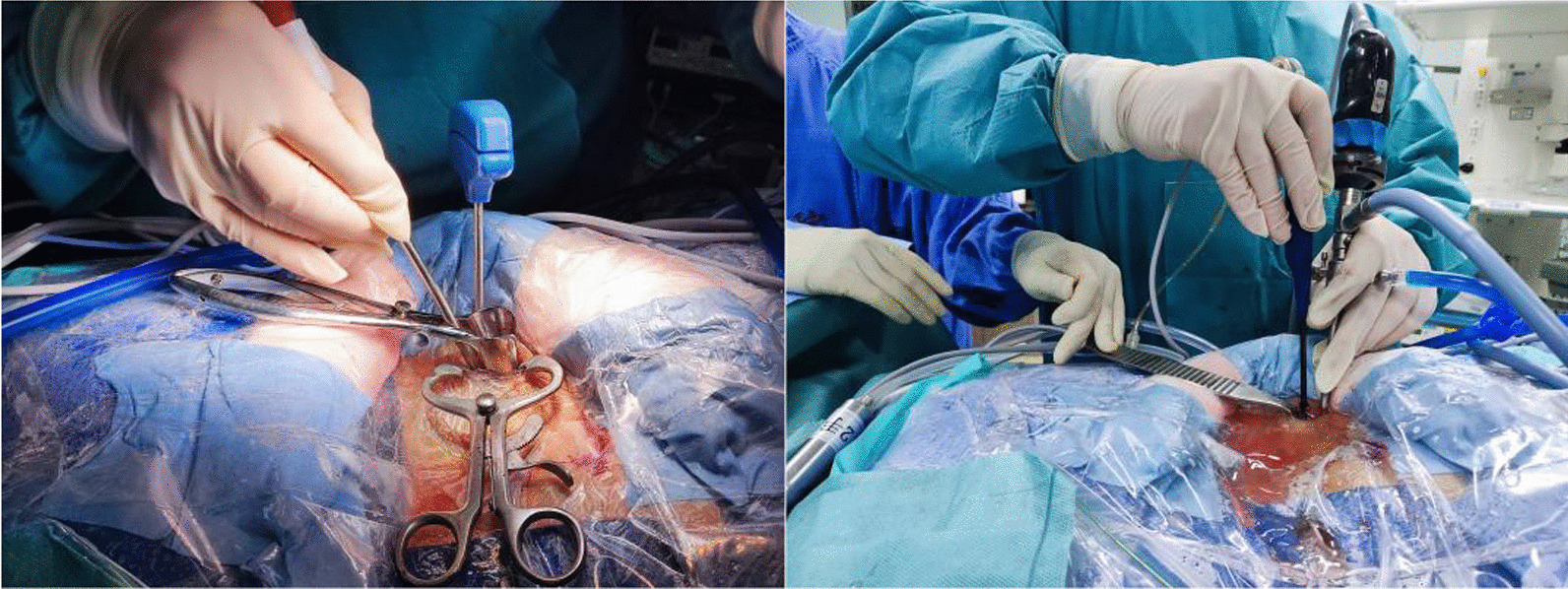
Intraoperative imaging of OSE technology

One-hole split endoscopy (OSE) is a new endoscopic surgery technique first proposed and applied by Professor Tengyue Zhu in 2019 [9, 10]. OSE technology has both working and observation channels, but both are located in the same incision, and each channel can move freely. It separates the single channel of the percutaneous transforaminal endoscopy discectomy(PTED), and integrates the two channels of the unilateral endoscopic technology into one channel. Its advantage is that the surgical working channel is not confined. Therefore, OSE not only has the convenience of PTED but also has the wide field of vision of unilateral biportal endoscopy (UBE) (Fig. 1).

Compared with open discectomy, OSE requires an intervertebral approach, which has been mastered by most spinal surgeons. Thus, OSE has a shorter learning curve for spinal surgeons planning to learn minimally invasive surgery. At present, OSE technology has been used by spinal surgeons to treat LDH in China. However, there is no systematic introduction or evaluation of its clinical efficacy for the treatment of LDH. Moreover, there is no clinical research comparing OSE with MED. The aim of the current retrospective cohort study is to evaluate the therapeutic effect of OSE and compare its efficacy with MED.

This is the first report evaluating the clinical outcomes of OSE for treating LDH. The current research has a positive impact on the promotion and application of OSE, which is a new minimally invasive and alternative treatment option for LDH.

## Methods

### Patient selection

This study compared the differences in clinical outcomes between OSE and MED during the 1-year follow-up after surgery, including life quality modification, pain control, and patient satisfaction. This was a retrospective control study that was conducted at Qilu Hospital of Shandong University. Between May 2019 and July 2022, we recruited 154 patients who underwent surgery for LDH; 68 consecutive patients underwent OSE by three surgeons, while 86 consecutive patients underwent MED by two surgeons.

### Inclusion and exclusion criteria

The inclusion criteria were as follows: (1) symptoms of sciatica related to LDH, (2) symptoms lasting more than 3 months and ineffective conservative treatment, and (3) symptom-related magnetic resonance (MR) or CT imaging data.

The exclusion criteria were as follows: (1) a history of lumbar surgery, (2) segmental instability (defined as > 3 mm translation or > 5° angulation), (3) comorbid tumorous or infectious conditions, (4) Meyerding grade II or higher spondylolisthesis, and (5) a protruded intervertebral disk with severe calcification that was difficult to remove by endoscopic surgery.

### Ethics and outcome assessment

The institutional review board of Qilu Hospital of Shandong University approved this study (KYLL-202309-31), and all patients submitted written informed consent forms. Data were gathered from the preoperative stage through the first year following surgery. The visual analog scale (VAS) score, modified MacNab score, and Oswestry Disability Index (ODI) score were assessed at 3-day, 1-month, 3-month, 6-month, and 12-month postoperative follow-ups used to measure pain severity, patient satisfaction, and improvement of dysfunction. To assess organ damage three days following surgery, the C-reactive protein (CRP) level was measured. EBL is calculated based on the formula proposed by Gross [11]. The number of days a patient is hospitalized after surgery (including the day of surgery) is used to determine the length of hospital stay. The formula suggested by Gross was used to determine the EBL. All minimally invasive surgical patients are routinely inserted with a drainage tube and urinary tube after surgery. On the second day after surgery, the drainage tube and urinary tube were removed under normal circumstances, and the X-ray, CT, and MRI were rechecked. When there are no abnormalities in the imaging results, the patient was encouraged to walk with wearing a waist circumference when the pain was tolerable. On the third day after surgery, patients can be discharged. After discharge, patients were advised to wear a waist circumference during physical activity. All patients were orally administered non steroidal anti-inflammatory drugs for 2 weeks after surgery. All patients were followed up at 1, 3, 6, and 12 months after surgery.

### Surgical techniques

#### OSE

The patient is lying prone on an arched cushion, which allows the opening of the vertebral lamina and the extending of the ligamentum flavum. The surgeon determines the surgical segment through intraoperative fluoroscopy and performs the surgical procedure by standing on the patient's right side. A skin incision approximately 1.5 cm long was made at the intersection of the inner edge of the pedicle and the intervertebral space, and the endoscope and surgical instruments were inserted through the incision. To create a working space, radiofrequency ablation was used to endoscopically cauterize the soft tissue. Then, the spine layer junction of the target intervertebral region was located, and partial laminotomy was performed using an electric drill to remove the lower edge of the upper vertebral plate and the inner edge of the articular process. The ligamentum flavum was removed with a Kerrison punch and radiofrequency probe, and then the annulus of the bulging intervertebral disk was dissected and exposed. Overgrown epidural arteries were carefully coagulated prior to discectomy to reduce the risk of hemorrhage. Kerrison punches and nucleus pulposus forceps were used to remove the burst fragments. Finally, nerve root decompression and dura mater pulsation were verified, a drain was placed, and the surgical wound was closed.

#### MED

Patients were positioned prone for surgical operations once general anesthesia was administered. C-arm fluoroscopy was used to confirm the target intervertebral space, and a Steinmann pin was inserted into the vertebral lamina at the surgical level. Next, an incision approximately 2 cm long was created at the Steinmann pin, and a series of dilators was used to expand the incision. Finally, a flexible table-mounted arm was used to secure a tubular retractor over the final dilator. In the working channel, a rigid endoscope with a 30° lens was inserted. A visual assistance system was used to perform the discectomy.

### Statistical analysis

SPSS version 26.0 software was used for statistical analysis. The independent samples *t* test, chi-square tests, and Mann‒Whitney *U* tests were used for intergroup comparisons; the paired *t* test was used for intragroup comparisons. Comparisons with values of *P* < 0.05 were considered statistically significant.

## Results

### Perioperative demographic parameters

**Fig. 2:**
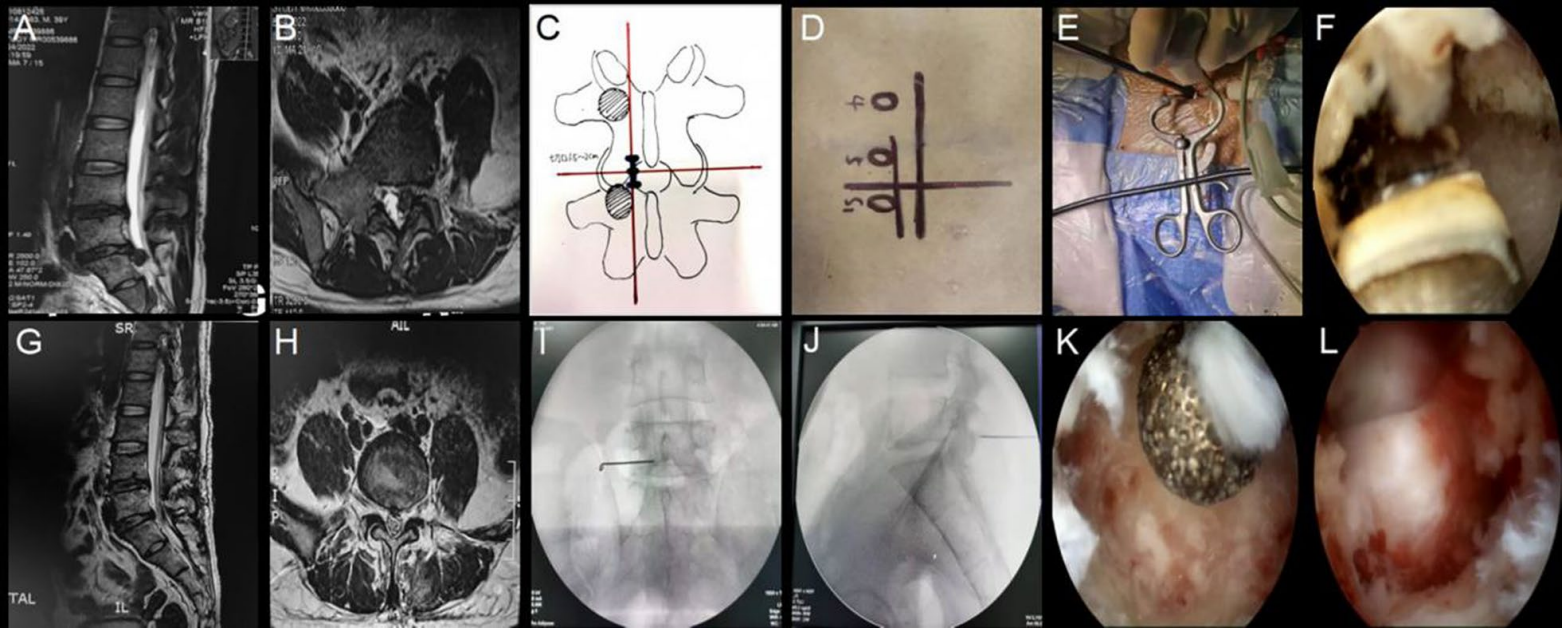
**A**, **B** Preoperative sagittal and axial magnetic resonance images in a 34-year-old male patient complaining of left radicular leg pain, showing L5–S1 disk herniation on the left side. **C**, **D** Surgical incision surface localization. **E** Establishing work channels. **F** Radiofrequency ablation exposed the lower edge of vertebral lamina and the inner edge of lower articular process. **G**, **H** Postoperative sagittal and axial magnetic resonance images made one day after surgery. **I**, **J** Kirschner wire anchoring positioning. K: Abrasive drill to thin the upper edge of the vertebral plate. **L** Removing the herniated intervertebral disk

One hundred fifty-four patients met the inclusion criteria, and neither the preoperative demographics nor the clinical features of the OSE (68 cases) and MED (86 cases) groups differed significantly (*P* > 0.05). The average age of the queue is 42.3 years old, with female patients accounting for 48.1% of the total. The most common types of intervertebral disk herniation are paracentral, with L4/5 and L5/S1 being the most common surgical levels. (Table 1). Typical case: A 34-year-old male underwent with OSE; VAS-back score was 4 points before surgery and 2 points, one point, and one point immediately after surgery, 1 months, and 3 months. VAS-leg score was 6 points before surgery and 2 points, one point, and one point immediately after surgery, 1 months, and 3 months (Fig. 2).

**Table 1:** Baseline demographic information of patients (OSE vs. MED)

Item	OSE (*n* = 68)	MED (*n* = 86)	*P* value
Age (years)	41.6 ± 10.7	42.9 ± 10.9	0.492
Gender (male/female)	38/30	42/44	0.385
BMI (kg/m^2^)	22.1 ± 2.9	22.5 ± 3.5	0.905
Disease duration (months)	11.8 ± 2.3	11.2 ± 2.5	0.106
Type of disk herniation			
Central	7 (10.3%)	11 (12.7%)	
Paracentral	55 (80.9%)	69 (80.2%)	0.776
Far lateral	6 (8.8%)	6 (7.0%)	
Surgical segment			
L3/4 or higher	5 (7.4%)	11 (12.8%)	
L4/5	41 (60.3%)	44 (51.2%)	0.469
L5/S1	22 (32.4%)	31 (36.0%)	
VAS score			
Back	5.8 ± 1.2	6.0 ± 1.9	0.219
Leg	7.0 ± 0.8	6.9 ± 0.9	0.922
ODI score	66.3 ± 14.6	67.7 ± 12.2	0.539

Continuous data were presented as mean with standard deviation. Categorical variables were presented as numbers with frequencies.* BMI* body mass index. *VAS* visual analog scale, *ODI* Oswestry Disability Index. *OSE* one-hole split endoscopy, *MED* microendoscopic discectomy

### Clinical outcome

The clinical outcome measurements are shown in Table 2. The surgical time was 72.6 ± 11.9 min in the OSE group and 75.6 ± 15.7 min in the MED group, with no statistically significant difference (*P* = 0.203). The estimated blood loss in the OSE group was 42.9 ± 12.2 mL, significantly lower than 56.1 ± 14.1 in the MED group (*P* < 0.001); The hospitalization time was 3.0 ± 0.9 days and 3.1 ± 1.1 days, respectively, with no statistically significant difference (*P* = 0.763); The incision length in the OSE group was 1.5 ± 0.3 cm, significantly lower than the MED group's 2.0 ± 0.5 (*P* < 0.001).

**Table 2:** Clinical outcome measurements (OSE vs. MED)

Parameter	OSE (*n* = 68)	MED (*n* = 86)	*P* value
Operation time (min)	72.6 ± 11.9	75.6 ± 15.7	0.203
Estimated blood loss (ml)	182.5 ± 52.5	270.4 ± 67.6	< 0.001*
Hospital stay (days)	3.0 ± 0.9	3.1 ± 1.1	0.763
Incision length (cm)	1.5 ± 0.3	2.0 ± 0.5	< 0.001*
CRP (mg/dl)	2.4 ± 0.8	2.7 ± 1.1	0.09

*Indicates significant difference. *CRP* amount of C-reactive protein at 48 h after surgery, *OSE* one-hole split endoscopy, *MED* microendoscopic discectomy

### Pain and life quality modification

**Fig. 3:**
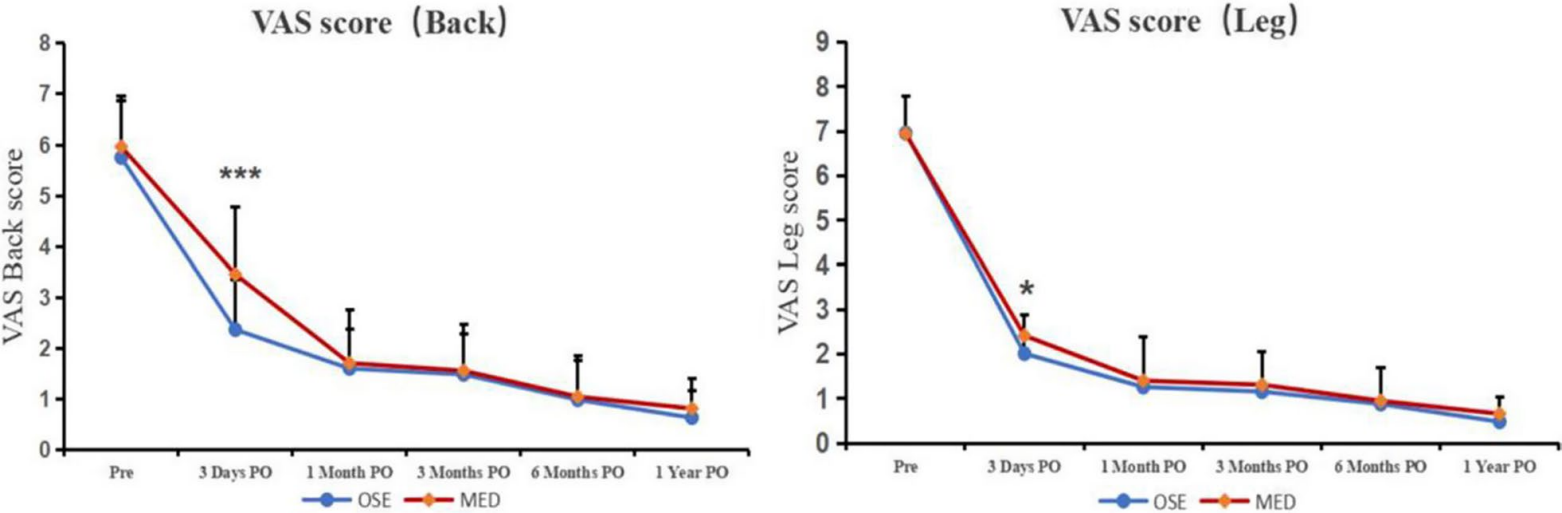
VAS score with follow-up time points (back and leg). *indicates significant difference. Significant differences were detected on the VAS back pain score on day 3 postoperatively (2.4 ± 1.0 scores in the OSE group, and 3.5 ± 1.3 scores in the MED group; *P* < 0.001) and VAS leg pain score (2.0 ± 0.9 scores in the OSE group, and 2.4 ± 1.0 scores in the MED group; *P* = 0.015)

There was no significant difference in preoperative VAS scores for the back and legs between the two groups. On the third day after surgery, there were significant differences in VAS back pain scores (2.4 ± 1.0 in the OSE group and 3.5 ± 1.3 in the MED group; *P* < 0.001) and VAS leg pain scores (2.0 ± 0.9 in the OSE group and 2.4 ± 1.0 in the MED group; *P = *0.015). At 1 month, 3 months, 6 months, and 12 months after surgery, there was no statistically significant difference in VAS scores between the two groups (*P* > 0.05) (Table 3; Fig. 3).

**Table 3:** VAS pain scores (back and leg) (OSE vs MED)

Groups	Preoperative	3 days PO	1 month PO	3 month PO	6 month PO	1 year PO
OSE (back)	5.8 ± 1.2	2.4 ± 1.0	1.6 ± 0.7	1.5 ± 0.8	1.0 ± 0.8	0.6 ± 0.5
MED (back)	6.0 ± 0.9	3.5 ± 1.3	1.7 ± 1.0	1.6 ± 0.9	1.1 ± 0.8	0.8 ± 0.6
*P* value	0.219	< 0.001*	0.586	0.785	0.677	0.069
OSE (leg)	7.0 ± 0.8	2.0 ± 0.9	1.3 ± 1.1	1.2 ± 0.9	0.9 ± 0.8	0.5 ± 0.6
MED (leg)	6.9 ± 0.9	2.4 ± 1.0	1.4 ± 0.9	1.3 ± 0.6	1.0 ± 0.8	0.7 ± 0.6
*P* value	0.922	0.015*	0.206	0.176	0.485	0.058

*PO* postoperative; *indicates significant difference. *VAS* visual analog scale, *ODI* Oswestry Disability Index. *OSE* one-hole split endoscopy, *MED* microendoscopic discectomy

**Fig. 4:**
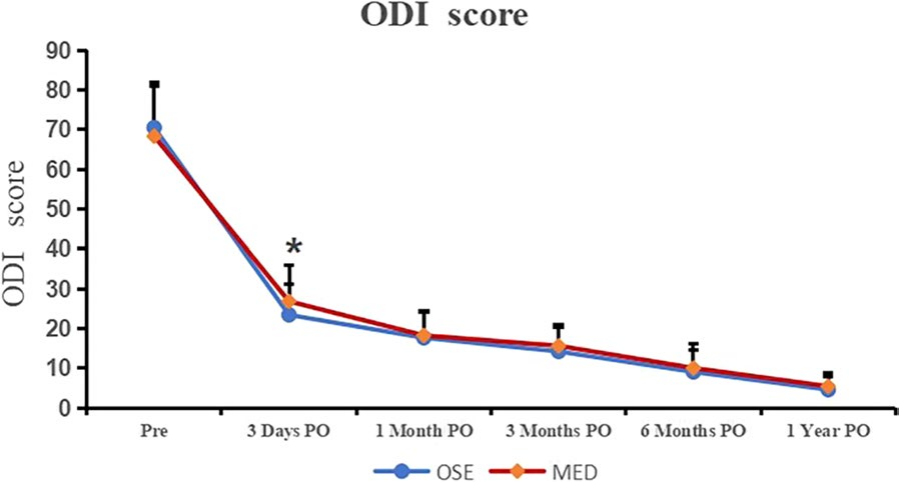
ODI score with follow-up time points. *indicates significant difference. Significantly better ODI scores were detected at 3 days postoperatively in the PTED group (23.4 ± 7.8 vs. 26.8 ± 9.1, *P* = 0.012)

The ODI score is shown in Table 4, and the trend of follow-up time is shown in Fig. 4. There was no significant difference in preoperative ODI scores between the OSE group (70.5 ± 11.3 points) and the MED group (68.3 ± 12.8 points; *P* = 0.571). The OSE group showed a significant improvement in ODI scores 3 days after surgery (23.4 ± 7.8 vs.26.8 ± 9.1, *P* = 0.012), and there was no significant difference at other follow-up time points. According to the MacNab standard, the satisfaction rates of the patients in the MED and OSE groups were 88.4% and 91.2%, respectively. There was no statistically significant difference in patient satisfaction between the two groups (*P* = 0.571; Table 5).

**Table 4:** ODI score (OSE vs. MED)

Time point	OSE (*n* = 68)	MED (*n* = 86)	*P* value
Preoperative	70.5 ± 11.3	68.3 ± 12.8	0.571
3 days PO	23.4 ± 7.8	26.8 ± 9.1	0.012*
1 month PO	17.7 ± 7.0	18.3 ± 6.1	0.564
3 months PO	14.2 ± 6.3	15.6 ± 5.4	0.065
6 months PO	9.0 ± 5.7	10.0 ± 6.3	0.326
1 year PO	4.5 ± 3.2	5.4 ± 3.3	0.102

*PO* postoperative; *indicates significant difference. *OSE* one-hole split endoscopy, *MED* microendoscopic discectomy

**Table 5:** Modified MacNab criteria (OSE vs. MED)

Groups	Cases	Excellent	Good	Fair	Poor	Rate (excellent and good)
OSE	68	38 (55.9%)	24 (35.3%)	5 (7.4%)	1 (1.5%)	62 (91.2%)
MED	86	45 (52.3%)	31 (36.0%)	7 (8.1%)	3 (3.5%)	76 (88.4%)
*χ*^2^ value		0.193	0.009	0.033		0.321
*P* value		0.660	0.923	0.856	0.630	0.571

### Postoperative complications

There were 5 cases (7.4%) of complications in the OSE group, and 13 cases (15.1%) in the MED group, with no significant difference (*P* = 0.136). The residual and recurrence rates were similar between the two groups (2.9% and 3.5%, respectively, *P* = 1.00), with the incidence of transient dysesthesia (2.9% and 5.8%, *P* = 0.465) and wound complications (1.5% and 3.5%, *P* = 0.630). There was no significant difference in the above parameters. (Table 6).

**Table 6:** Postoperative complications (OSE vs MED)

Parameter	OSE (*n* = 68)	MED (*n* = 86)	*P* Value
Dural tear	0	2	0.504
Transient dysesthesia	2	5	0.465
Poor wound healing	1	3	0.630
Residue or recurrence	2	3	1.000
Total	5	13	0.136
Reoperation	2	4	0.694

^*^Indicates significant difference. *OSE* one-hole split endoscopy, *MED* microendoscopic discectomy

## Discussion

The aim of the present study was to retrospectively compare MED and OSE for LDH. To the best of our knowledge, no previous studies have compared OSE with MED. MED is regarded as one of the standard minimally invasive treatment options and is routinely performed to treat LDH. Therefore, MED was used as a reference to evaluate the efficiency and safety of OSE. Our results demonstrated that compared with MED, OSE is a new alternative treatment option for LDH that can achieve similar and satisfactory clinical outcomes. Furthermore, OSE has many advantages, including less EBL and a smaller incision.

With the development of endoscopic technology, endoscopic lumbar discectomy has become popular, and the technology has been continuously improved [12, 13]. In recent years, the representative endoscopic techniques mainly include MED, PTED and UBE, which have achieved satisfactory clinical results [14, 15]. However, the common feature of all surgical methods is that the light source and instruments must be operated through fixed channels, which restricts surgeons from choosing a wider surgical field of vision to some extent. Spinal surgeons expect that endoscopy has a wide field of vision and freedom of movement, similar to open surgery, to improve efficiency and reduce trauma and complications. Various endoscopic procedures have advantages and disadvantages [16–21]. MED is similar to open operation in that air medium is used, but blood stains often cause blurred vision. MED and PTED require special instruments in the operation. PTED is performed in a narrow channel, which increases the risk of nerve injury. In the working channel of PTED, the surgical instruments can be moved freely in all directions, and surgeons can accurately point to the target area. UBE has a wide field of vision. However, due to biportal working channels and limitations in device implantation, UBE causes more tissue damage. In addition, multiple entrances and the lack of a closed joint space make exchanging and colocating instruments more technically challenging.

Based on the desirability of no fixed channel, one-hole split scope (OSE) not only allows free instrument movement as PTED but also has a wide field of vision as UBE. It allows random switching between water and air media [22, 23]. An incision of approximately 1.5 cm can ensure sufficient water pressure, with the shortest surgical path to the operation area and clear vision.

In the present study, the VAS and ODI scores of the two groups were both significantly improved when compared with those before surgery. At the 3-day follow-up, the improvements in the postoperative back and leg VAS scores and ODI score in the OSE group were significantly better than those in the MED group. These factors can be used to explain this. First, in the MED group, prolonged tubular retraction may cause denervation and ischemia of the paraspinal muscle, thereby leading to muscular atrophy and discomfort after the operation [24]. Second, unlike MED, OSE is performed with continuous irrigation of saltwater, which has certain benefits. For instance, it has been suggested that saline irrigation might lessen the release of inflammatory mediators that lead to postoperative back discomfort [25, 26].

The EBL of the OSE group was significantly lower than that of the MED group. We believe that sufficient water pressure can compress small blood vessels, and low-temperature physiological saline can also constrict small blood vessels [7, 27]. In addition, the tubular retractor used in MED can only be placed outside the spinal canal, which cannot effectively control hemorrhage in the spinal canal [8, 28]. The operation duration of the MED group was longer, but there was no significant difference between the two groups. In the air medium, severe bleeding seriously affects the recognition of the visual field under the microscope and increases the probability of complications such as nerve damage, thereby reducing work efficiency and increasing surgical time [29, 30].

In terms of the incidence of complications, two patients in the MED group exhibited dural tears. One of the patients recovered and was left the hospital after lying in bed and receiving conservative treatment. Another patient had sustained wound nonunion due to cerebrospinal fluid leakage and finally underwent secondary surgery. We believe that the perfusion pressure of saline solution can establish a space between the dura mater and the ligamentum flavum, thus facilitating the biting off of the ligamentum flavum without damaging the dura mater [26]. There were 5 patients with residue or recurrence, 3 in the MED group and 2 in the OSE group, and there was no significant difference between the two groups. We consider that because the tubular retractor is placed outside the spinal canal in MED, incomplete removal of the nucleus pulposus is caused by limited vision. Transitional dysesthesia is more prevalent in the MED group, likely as a result of nerve root traversal and dural sac retraction [25, 31, 32]; additionally, the likelihood of wound problems is greater because of the wider incision needed for the MED approach.

This study had several limitations. First, there were no radiologic results, such as lumbar spine stability or neighboring segment deterioration. Second, a retrospective design was used in the study. Prospective, multicenter trials with larger sample sizes are needed in the future to compare the long-term clinical results of OSE with those of other endoscopic surgeries.

## Conclusion

Compared with MED, OSE is a new alternative treatment option for LDH that can achieve similar and satisfactory clinical outcomes. Furthermore, OSE has many advantages, including less EBL and a smaller incision. Further clinical studies are needed to confirm the effectiveness of OSE.
